# Temperature‐driven modulation of factors influencing full‐Marathon time in male recreational runners

**DOI:** 10.14814/phy2.71096

**Published:** 2026-09-08

**Authors:** Koshiro Inoue, Akihiko Yamaguchi, Yoshiharu Nabekura, Toru Ishihara, Takemune Fukuie

**Affiliations:** ^1^ Center for Education in Liberal Arts and Sciences, School of Rehabilitation Sciences Health Sciences University of Hokkaido Tobetsu Hokkaido Japan; ^2^ Institute of Health and Sport Sciences University of Tsukuba Tsukuba Ibaraki Japan; ^3^ Graduate School of Human Development and Environment Kobe University Kobe Hyogo Japan; ^4^ Center for Education in Liberal Arts and Sciences, School of Nursing and Social Services Health Sciences University of Hokkaido Tobetsu Hokkaido Japan

**Keywords:** full‐marathon, performance determinants, recreational runner, temperature differences

## Abstract

The weather during full‐marathon events is expected to become warmer due to global warming, making heat preparations a key challenge for runners. To gain insight into this challenge, we examined whether associations between full‐marathon finish time and selected factors differed across the Tsukuba Marathon 2023 and 2022, and the Hokkaido Marathon 2023, which represent optimal, moderate, and extreme temperatures, respectively. Questionnaire‐based measurements of full‐marathon personal best (PB), age, running experience (career), body mass index (BMI), weekly running distance and frequency, longest running distance, and finish time were analyzed using hierarchical multiple regression analysis, followed by simple slope analyses for significant interactions. The analysis included 1938 male runners. Event‐based ambient temperature conditions independently explained approximately 11% of the variance in finish time. Temperature conditions significantly modulated the relationships between finish time and PB, age, career, and BMI, whereas no such effect was observed for running distance, frequency, and longest run. Specifically, the temperature‐related increase in finish time tended to be greater in runners characterized by faster PB time, older age, lower career values, and higher BMI. Running capacity, age, running experience, and BMI may serve as key targets in strategies to counteract heat‐related marathon performance decline in male recreational runners.

## 1 INTRODUCTION

The optimal air temperature (Ta) for recreational runners in a full‐marathon (hereinafter referred to as “marathon”) is 3.0°C–11.0°C (Arielle, 2025; El Helou et al., 2012). However, Climate Central, a US climate research organization, has reported that because of global warming, opportunities to run a marathon in optimal temperatures will decrease going forward (Arielle, 2025). In other words, more races are likely to be held in hotter conditions. For example, among recreational runners, the Tokyo Marathon is considered to offer the highest likelihood of competing under optimal Ta conditions, with the probability projected to decline from 79% and 81% in 2025 to 72% and 74% by 2045 for the male and female groups, respectively (Arielle, 2025). Although recreational runners' motivations for participating in races vary depending on factors such as experience, age, and family circumstances, pursuing personal goals and embracing new challenges are significant motivators (Partyka & Waskiewicz, 2024). Therefore, the factors influencing the finish time of a marathon, that is the determinants of marathon performance, may be considered one of the major concerns among recreational runners. Given the anticipated increase in races held in weather conditions above optimal temperatures, it is necessary to consider the impact of heat when examining the determinants of marathon performance.

To date, a large body of research has been vigorously focused on predicting marathon finish times or race pace based on various variables. It is well known that marathon performance can be determined by physiological parameters of aerobic fitness, such as maximal oxygen uptake (V̇O_2max_), lactate/ventilatory threshold, and running economy (Barnes & Kilding, 2015; Esteve‐Lanao et al., 2021; Foster, 2007; Hagan et al., 1981; Joyner, 1991; Sjödin & Svedenhag, 1985; Takeshima & Tanaka, 1995; Tanaka & Matsuura, 1984). Furthermore, marathon finish times and race pace are influenced and determined by various factors, including training behaviors (e.g., weekly running distance, running speed during training, running distance per workout, and number of training sessions per week) (Bale et al., 1985; Barandun et al., 2012; Dotan et al., 1983; Hagan et al., 1981; Hagan et al., 1987; Nikolaidis & Knechtle, 2024; Schmid et al., 2012; Tanda, 2011; Tanda & Knechtle, 2013; Yamaguchi et al., 2023), anthropometric parameters (e.g., body fat percentage, body mass index (BMI), and calf circumference) (Barandun et al., 2012; Dotan et al., 1983; Hagan et al., 1987; Keogh et al., 2020; Salinero et al., 2017; Schmid et al., 2012; Tanda & Knechtle, 2013), somatotype (Bale et al., 1985), sex (Keogh et al., 2020), age (Dotan et al., 1983; Hagan et al., 1981; Keogh et al., 2020; Takeshima & Tanaka, 1995), and history of running experience (Bale et al., 1985; Keogh et al., 2020). Also, best records over specific distances (e.g., 10 km, half marathon) have been identified as factors influencing marathon times when combined with other factors (Noakes et al., 1990; Salinero et al., 2017; Slovic, 1977). Using these factors, a diverse range of prediction equations has been developed to estimate marathon finish times and race pace, and various combinations of predictors have been reported in previous studies (Alvero‐Cruz et al., 2020).

Within these predictive factors, running capacity, age, and sex have been shown to modulate the degree to which temperature impacts marathon performance. For example, the impact of rising temperatures on marathon performance may depend on running capacity. Evidence on this is conflicting, with some studies reporting greater performance declines under hot Ta conditions in slower runners (Ely, Cheuvront, Roberts, & Montain, 2007; Montain et al., 2007; Vihma, 2010) and others in faster runners (Gasparetto & Nesseler, 2020). The thermal impact on marathon performance as mediated by running capacity remains unclear. For age, Knechtle, Mcgrath, et al. (2021) have reported that increasing Ta is linked to slower finish times, and this tendency is reinforced among men aged 30–64 and women aged 40–64 (Knechtle, Mcgrath, et al., 2021). Hot weather also appears to influence marathon performance in a sex‐dependent manner, with women showing a smaller decline than men (Vihma, 2010), although a few reports suggest there is no gender difference in the degree/pattern of reduction in race times associated with increasing Ta (El Helou et al., 2012; Knechtle, Valero, et al., 2021). In addition to these factors, anthropometric characteristics (Dennis & Noakes, 1999; Selkirk & Mclellan, 2001), aerobic fitness level (Lisman et al., 2014; Selkirk & Mclellan, 2001), and aerobic training (Cheung et al., 2000; Lorenzo et al., 2010) are considered potential contributors related to heat intolerance or acclimation. Hence, we hypothesized that the decline in marathon performance associated with rising Ta may vary depending on factors such as running ability, biological age, training status, and body characteristics. However, no studies have systematically examined how these predictive factors interact with the Ta‐related differences. Consequently, it remains unclear which factors are relatively sensitive to temperature variation and are significantly critical when formulating effective heat‐mitigation strategies.

To address this issue, this study used a questionnaire survey on factors that may influence marathon performance in relation to temperature changes in two marathon events held in different seasons: the Tsukuba Marathon is held in November, when optimal temperatures are anticipated, and the Hokkaido Marathon is held in August, when high temperatures are anticipated. A hierarchical multiple regression analysis including interaction term was performed to identify factors whose effects on marathon finish time are modulated by race conditions, particularly Ta‐related differences.

## 2 MATERIALS AND METHODS

### 2.1 Ethical approval

This study was conducted in accordance with the Declaration of Helsinki and approved by the Institutional Ethics Committee of Health Sciences University of Hokkaido (approved no. 22R191189, 23R212216).

### 2.2 Marathons

A questionnaire survey was conducted for participants in the Tsukuba Marathon 2022 (T22, November 13) and 2023 (T23, November 26), and the Hokkaido Marathon 2023 (H23, August 27). The survey requests were distributed to all T22 and T23 runners, and randomly to 5000 H23 runners, with the consent of each organizing committee. Both races set a time limit of 6 h to finish; T22 and T23 started at 9:00 a.m., and H23 started at 8:30 a.m. The T22 and T23 course was mostly flat throughout with approximately 11 m of elevation changes, whereas that of H23 had an elevation difference of approximately 30 m within the first 10 km but was largely flat thereafter. An overview of the weather conditions for each race was reported as follows by the Japan Meteorological Agency. For T22, the mean Ta and relative humidity (Rh) during the race were 20.6°C and 69.6%, respectively. The lowest Ta was 14.2°C with 93% Rh at 9:00, while the highest Ta was 23.4°C with 55% Rh at 12:40. For T23, the mean Ta and Rh during the race were 6.5°C and 94.0%, respectively. The lowest Ta was 5.3°C with 90% Rh at 9:00, while the highest Ta was 7.6°C with 93% Rh at 14:10–14:20. For H23, the mean Ta and Rh during the race were 28.2°C and 77.4%, respectively. The lowest Ta was 24.0°C with 92% Rh at 14:00, while the highest Ta was 30.6°C with 72% Rh at 12:00. As direct wet‐bulb globe temperature (WBGT) measurements were not available, a simplified WBGT (sWBGT) was estimated from the mean Ta and Rh during the race using a validated approximation formula recommended by the American College of Sports Medicine (ACSM) (ACSM, 1984). The equation was:

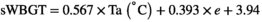

where 𝑒 denotes water vapor pressure (hPa), calculated as follows:

$$\text{𝑒}=\left(\mathrm{Rh}/100\right)\times 6.105\times \exp\;\left(17.27\times \mathrm{Ta}/\left(237.7+\mathrm{Ta}\right)\right)$$

The estimated sWBGTs were 22.2°C, 11.2°C, and 31.5°C for T22, T23, and H23, respectively. This approach provides a practical estimate of environmental heat stress in large‐scale outdoor endurance events.

### 2.3 Participants

**FIGURE 1:**
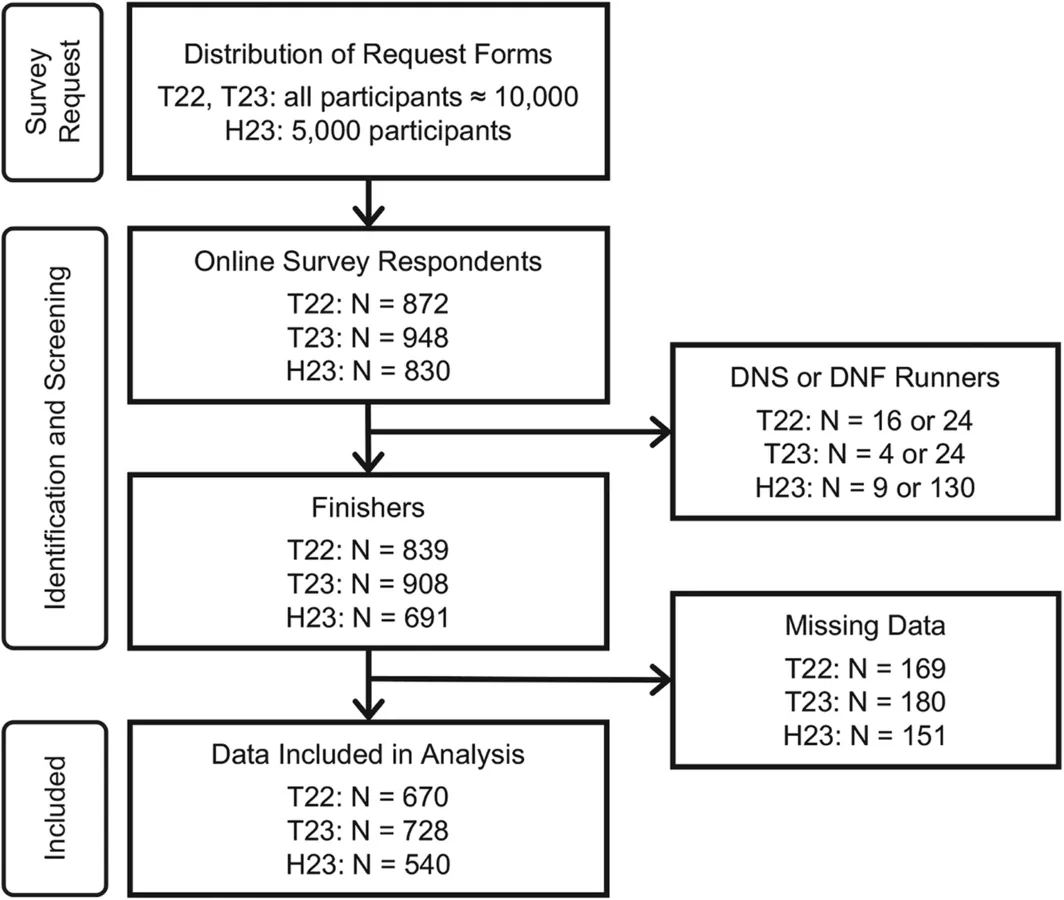
Overview of runner selection process. DNF, did not finish; DNS, Did not start.

A priori power analysis using G*Power (version 3.1) (Faul et al., 2009) indicated that a minimum of 1022 datasets was required to achieve our primary objective of detecting interaction effects in a hierarchical regression model (*f* ^2^ = 0.02, *α* = 0.05, power = 0.80, 29 predictors, 18 interaction terms). As prior studies did not provide a reliable estimate of the expected effect size, we assumed a small effect size (*f* ^2^ = 0.02) based on Cohen (1988). Given the inclusion of multiple covariates, the effect of the predictor of interest and its incremental contribution to explained variance was expected to be small; therefore, this conservative estimate was adopted. In addition, because of sex‐specific differences in the temperature–performance relationship (Keogh et al., 2020; Vihma, 2010) and the potential difficulty in obtaining the required sample size of female runners, only male runners were included in the present study. Figure 1 shows an overview of the runner selection process and the number of recruitments. Runners who had an interest in the survey voluntarily completed a questionnaire before the start of the race and participated in the study (T22: *N* = 872, T23: *N* = 948, H23: *N* = 830). The participants were provided with written information regarding the aims and possible risks associated with the survey, and informed consent was obtained electronically through the online survey before participation. To meet the study objectives, participants who did not finish the race or who had missing data were excluded. Most of the missing data were full‐marathon personal best (PB), which is a questionnaire item included as an indicator of running capacity. Finally, a total of 1938 datasets were included (T22: *N* = 670; T23: *N* = 728, H23: *N* = 540). The final sample size exceeded the requirement estimated by the a priori power analysis.

### 2.4 Questionnaire survey

Runners who agreed to complete the full survey accessed an online questionnaire through a QR code and responded to it. The questionnaire items included marathon entry number, sex, PB over the last 3 years for T22, or last 1 year for T23 and H23, age, years of running experience (career), weight, height, and training status during the 3 months prior to the races, which corresponds to the period from September to November for T22 and T23 and from June to August for H23. It should be noted that, in T22, given the two‐year suspension of marathon race due to the COVID‐19 pandemic, PBs from the preceding 3 years, including the pre‐pandemic year, were collected. Training status was specifically investigated by monthly running distance, longest running distance per workout (hereinafter “longest run”), and average weekly running frequency (hereinafter “running frequency”). Factors other than sex and running frequency were obtained from free descriptive answers. The runners reported their sex as male or female and their running frequency according to the following categories: (1) less than 1 time/week, (2) 1–2 times/week, (3) 3–4 times/week, and (4) 5 or more times/week. All questions had the option to leave them unanswered. BMI was calculated using the formula weight (kg) divided by height squared (m^2^). Since the number of days in the three‐month period preceding each race varied, weekly running distance (hereinafter “running distance”) was estimated by dividing the total monthly running distance covered during that period by the number of days and converting this value to a weekly equivalent. The net finish time (hereinafter “finish time”) of each runner was obtained from the official website based on their entry numbers. For ease of analysis, the three marathons were ordered by temperature from lowest to highest and classified as the following event variables: T23 = Event 1 (mean Ta: 6.5°C; sWBGT: 11.2°C), T22 = Event 2 (mean Ta: 20.6°C; sWBGT: 22.2°C), H23 = Event 3 (mean Ta: 28.2°C; sWBGT: 31.5°C). Running frequency and event were treated as categorical variables.

### 2.5 Statistical analysis

All statistical analyses were performed using R (Version 4.4.1) with significance levels set at *α* = 0.05. The surveyed variables are summarized by descriptive statistics. The data are shown as mean ± standard deviation (SD) plus range for numeric data or number (*N*) and percentage of the total (%) for categorical data. Since not all data were normally distributed in the Shapiro–Wilk test (*p* < 0.001), differences between races were compared using the Kruskal–Wallis test with Dunn's post‐hoc test, or the pairwise chi‐squared test with Holm correction. Regression analysis was performed to examine the significance of the univariate association between finish time and each variable. For inferential statistics, a hierarchical multiple regression (HMR) analysis was conducted to determine the predictor variables contributing to the outcome (finish time), considering the interaction with Event, that is with temperature difference. In the first model, variables excluding Event were entered (Model 1: finish time~PB + age + career + BMI + running distance + longest run + running frequency). In the second model, Event was introduced into Model 1 to clarify the additional contribution of temperature changes on finish time (Model 2: finish time~PB + age + career + BMI + running distance + longest run + running frequency + Event). Event was a categorical variable with three levels ordered by temperature (1: T23, reference; 2: T22; 3: H23). In the third model, the interaction terms between Event and each other variable were introduced into Model 2 to examine the variables modified by Events in the prediction of finish time (Model 3: finish time~(PB + age + career + BMI + running distance + longest run + running frequency) × Event). Before calculating the interaction terms, the continuous variables were centered to reduce multicollinearity between the main effects and interaction terms. Model improvement was assessed with the change of *R* ^2^ (Δ*R* ^2^) and the *F*‐test between the Models. Moreover, a type III analysis of variance (ANOVA) was performed on Model 3 to clarify the unique contribution of each variable and interaction term in predicting finish time after adjustment for all other predictors. Simple slope analysis was conducted for the interaction term where significance was confirmed in the type III ANOVA using estimated marginal trends. Moreover, pairwise comparisons of the slopes between Events were performed with Tukey adjustment for multiple comparisons. The significant interaction effects were visualized by plotting estimated marginal means of finish time across a range of predictor values (−2 SD to +2 SD) to facilitate interpretation. Prior to the regression analyses, all continuous variables were *z*‐standardized. Categorical predictors were dummy‐coded and entered without standardization. Therefore, coefficients for continuous predictors can be interpreted as standardized regression coefficients (*β*), whereas coefficients for categorical predictors represent differences relative to the reference category on the standardized outcome scale.

## 3 RESULTS

### 3.1 Characteristics of subjects

Demographic information for the runners ultimately included is summarized in Table 1. Significant differences between Events were observed for finish time, PB, career, BMI, running distance, longest run, and running frequency (*p* < 0.05). The finish times were shortest for T23 runners, followed by T22 and H23 runners (*p* < 0.001), while the PBs were shortest for T22 runners, followed by T23 and H23 runners (*p* < 0.001). Compared with H23 runners, T23 and T22 runners had shorter careers (*p* = 0.002), lower BMIs (*p* < 0.001), longer running distances (*p* < 0.001), and higher running frequency (*p* < 0.001). Furthermore, the longest runs were greatest for T22 runners, followed by T23 and H23 runners (*p* < 0.001). By contrast, there was no significant difference between the Events for age.

**TABLE 1:** Characteristics of subjects.

Variable	Overall	a: T23	b: T22	c: H23	*p*‐value	Post‐hoc
*N*	1938	728	670	540		
Finish time (min)	242.9 ± 48.2 [148.7–369.2]	223.3 ± 39.1 [148.7–353.0]	233.8 ± 43.7 [155.2–369.2]	280.7 ± 43.4 [160.4–364.6]	<0.001	a<b, a<c, b<c
PB (min)	230.2 ± 42.9 [133.0–378.0]	227.2 ± 42.0 [149.0–378.0]	219.7 ± 38.8 [133.0–357.0]	247.3 ± 43.8 [151.0–359.0]	<0.001	a>b, a<c, b<c
Age (years)	51.3 ± 8.9 [22.0–78.0]	51.3 ± 8.7 [23.0–78.0]	51.4 ± 8.4 [22.0–76.0]	51.1 ± 9.9 [22.0–75.0]	0.852	
Career (years)	12.3 ± 8.3 [1.0–61.0]	11.6 ± 8.2 [1.0–61.0]	11.9 ± 7.3 [1.0–43.0]	13.6 ± 9.5 [1.0–49.0]	0.002	a<c, b<c
BMI (kg/m^2^)	21.4 ± 2.0 [15.1–30.8]	21.2 ± 1.9 [17.1–30.8]	21.3 ± 1.9 [15.1–29.2]	21.9 ± 2.1 [15.6–30.1]	<0.001	a<c, b<c
Running distance (km/week)	45.3 ± 23.9 [0.3–189.2]	45.1 ± 22.3 [1.5–156.1]	46.8 ± 22.0 [3.8–158.9]	43.8 ± 27.8 [0.3–189.2]	<0.001	a>c, b>c
Longest run (km/time)	31.6 ± 13.7 [6.7–139.0]	31.8 ± 12.3 [9.0–120.0]	33.5 ± 13.9 [10.0–139.0]	29.0 ± 14.8 [6.7–128.0]	<0.001	a<b, a>c, b>c
Running frequency (times/week)						
Group 1: <1	52 (2.7)	16 (2.2)	11 (1.6)	25 (4.6)	<0.001	a>c, b>c
Group 2: 1–2	500 (25.8)	160 (22.0)	147 (21.9)	193 (35.7)		
Group 3: 3–4	799 (41.2)	319 (43.8)	274 (40.9)	206 (38.1)		
Group 4: ≥5	587 (30.3)	233 (32.0)	238 (35.5)	116 (21.5)		

*Note*: Values are presented as mean ± SD [Range] for numeric data, and as *N* (%) for categorical data. For running frequency, the pairwise chi‐squared test with Holm correction was performed to compare the values of each race. For the other variables, the Kruskal–Wallis test with Dunn's post‐hoc test was performed to compare the values of each event.

Abbreviations: BMI, body mass index; Career, years of running experience; PB, personal best.

### 3.2 Association between finish time and predictor variables

The relationship between finish time and the predictor variables examined by univariate linear regression analysis is shown in Table S1. A significant association with finish time was confirmed for all variables. PB (*p* < 0.001), age (*p* < 0.001), career (*p* = 0.006), BMI (*p* < 0.001), and Event (*p* < 0.001) had significantly positive associations with finish time, whereas a significant negative association was found between finish time and running distance (*p* < 0.001), longest run (*p* < 0.001), or running frequency (*p* < 0.001).

### 3.3 Hierarchical multiple regression (HMR) analysis

Summary of the HMR models and the associated regression coefficients are provided in Tables 2 and 3, respectively. The first model significantly explained finish time (*F* = 523.0, *p* < 0.001, Table 2) with an *R* ^2^ of 0.709. Among the included variables, PB (*β* = 0.66, *p* < 0.001), age (*β* = 0.12, *p* < 0.001), BMI (*β* = 0.11, *p* < 0.001), career (*β* = 0.05, *p* < 0.001), and longest run (*β* = −0.04, *p* = 0.003) showed significant predictive effects on finish time, whereas running distance (*p* = 0.057) was not significant (Table 3). For the categorical variable running frequency, runners in Groups 2–4 showed significantly shorter finish time compared with the reference runners in Group 1 (Group 2: *β* = −0.31, Group 3: *β* = −0.48, Group 4: *β* = −0.56, all *p* < 0.001, Table 3). In Model 2, the addition of the Event effect to Model 1 resulted in a significant increase in explained variance by 10.7% (*F* of Δ*R* ^2^ = 556.8, *p* < 0.001, Table 2). In this model, PB (*β* = 0.58, *p* < 0.001), age (*β* = 0.14, *p* < 0.001), running distance (*β* = −0.14, *p* < 0.001), and BMI (*β* = 0.08, *p* < 0.001) were significant predictors, whereas career and longest run were no longer significant (Table 3). For running frequency, finish time remained significantly shorter in Groups 2–4 than in the reference Group 1 (all *p* < 0.001, Table 3). For Event, relative to the reference Event T23, Events T22 (*β* = 0.33, *p* < 0.001) and H23 (*β* = 0.86, *p* < 0.001) were associated with significantly longer finish time. When the reference category was changed to Event T22, significantly longer finish time was observed in Event H23 (*β* = 0.53, *p* < 0.001). In short, the regression coefficients of Event indicated a stepwise increase in finish time from Event T23 to T22 and from Event T22 to H23 (T23 <T22 <H23).

**TABLE 2:** Summary of hierarchical multiple regression models.

Model fit	Model 1	Model 2	Model 3
*R* ^2^	0.709	0.816	0.825
Adj *R* ^2^	0.708	0.815	0.822
*F*	523.0	775.9	309.0
*p*‐value	<0.001	<0.001	<0.001
AIC	12648.5	11768.3	11711.8
Versus Model 1			
Δ*R* ^2^		0.107	0.115
*F* of Δ*R* ^2^		556.8	62.5
*p*‐value		<0.001	<0.001
Versus Model 2			
Δ*R* ^2^			0.009
*F* of Δ*R* ^2^			5.2
*p*‐value			<0.001

*Note*: Model 1: finish time~PB + age + career + BMI + running distance + longest run + running frequency; Model 2: finish time~PB + age + career + BMI + running distance + longest run + running frequency + Event; Model 3: finish time~(PB + age + career + BMI + running distance + longest run + running frequency) × Event.

Abbreviations: Adj *R* ^2^, adjust *R* squared; AIC, the akaike information criterion; *R* ^2^, *R* squared.

**TABLE 3:** Model coefficients.

Variables	*B*	SE	*β*	*t*‐value	*p*‐value	Adjusted GVIF
Model 1: Base model						
Intercept	6.83	9.18	0.45	0.74	0.457	
PB	0.75	0.02	0.66	40.14	<0.001	1.35
Age	0.63	0.07	0.12	8.56	<0.001	1.10
Career	0.28	0.08	0.05	3.63	<0.001	1.07
BMI	2.72	0.33	0.11	8.24	<0.001	1.10
Running distance	−0.08	0.04	−0.04	−1.90	0.057	1.61
Longest run	−0.14	0.05	−0.04	−2.95	0.003	1.10
Running frequency Group 2	−14.75	3.83	−0.31	−3.85	<0.001	1.13
Running frequency Group 3	−23.11	3.90	−0.48	−5.92	<0.001	−
Running frequency Group 4	−26.97	4.28	−0.56	−6.30	<0.001	−
Model 2: Model 1 + Main effects of Event						
Intercept	20.35	7.35	−0.07	2.77	0.006	
PB	0.66	0.02	0.58	43.04	<0.001	1.39
Age	0.78	0.06	0.14	13.29	<0.001	1.11
Career	0.04	0.06	0.01	0.59	0.557	1.08
BMI	1.98	0.26	0.08	7.51	<0.001	1.11
Running distance	−0.29	0.03	−0.14	−8.80	<0.001	1.65
Longest run	−0.06	0.04	−0.02	−1.64	0.102	1.10
Running frequency Group 2	−11.28	3.06	−0.23	−3.69	<0.001	1.14
Running frequency Group 3	−14.79	3.12	−0.31	−4.74	<0.001	−
Running frequency Group 4	−14.80	3.43	−0.31	−4.31	<0.001	−
Event T22	15.74	1.12	0.33	14.08	<0.001	1.04
Event H23	41.42	1.24	0.86	33.37	<0.001	−
Model 3: Model 2 + Interaction between each variable and Event						
Intercept	242.47	5.39	−0.01	44.95	<0.001	
PB	0.70	0.03	0.62	27.88	<0.001	2.33
Age	0.54	0.10	0.10	5.57	<0.001	1.88
Career	0.14	0.10	0.02	1.42	0.156	1.79
BMI	0.44	0.44	0.02	1.01	0.314	1.89
Running distance	−0.22	0.06	−0.11	−3.87	<0.001	2.91
Longest run	−0.06	0.07	−0.02	−0.87	0.383	1.96
Running frequency Group 2	−13.52	5.44	−0.28	−2.49	0.013	1.94
Running frequency Group 3	−18.13	5.49	−0.38	−3.30	0.001	−
Running frequency Group 4	−18.53	5.93	−0.38	−3.13	0.002	−
Event T22	11.58	8.30	0.24	1.40	0.163	6.67
Event H23	39.11	6.97	0.81	5.61	<0.001	−
PB × Event T22	0.02	0.04	0.02	0.61	0.543	1.77
PB × Event H23	−0.14	0.04	−0.12	−3.86	<0.001	−
Age × Event T22	0.33	0.14	0.06	2.37	0.018	1.44
Age × Event H23	0.40	0.14	0.07	2.85	0.004	−
Career × Event T22	0.20	0.15	0.03	1.31	0.192	1.39
Career × Event H23	−0.46	0.14	−0.08	−3.23	0.001	−
BMI × Event T22	1.46	0.63	0.06	2.31	0.021	1.44
BMI × Event H23	2.90	0.64	0.12	4.54	<0.001	−
Running distance × Event T22	−0.18	0.08	−0.09	−2.18	0.029	2.20
Running distance × Event H23	−0.09	0.08	−0.04	−1.10	0.271	−
Longest run × Event T22	−0.05	0.09	−0.01	−0.53	0.599	1.49
Longest run × Event H23	0.07	0.09	0.02	0.78	0.435	−
Running frequency Group 2 × Event T22	3.67	8.39	0.08	0.44	0.662	2.50
Running frequency Group 2 × Event H23	1.94	6.99	0.04	0.28	0.781	−
Running frequency Group 3 × Event T22	4.64	8.44	0.10	0.55	0.582	−
Running frequency Group 3 × Event H23	5.02	7.17	0.10	0.70	0.484	−
Running frequency Group 4 × Event T22	7.41	8.99	0.15	0.82	0.410	−
Running frequency Group 4 × Event H23	5.86	8.13	0.12	0.72	0.471	−

Abbreviations: Adjusted GVIF, degree‐of‐freedom‐adjusted generalized variance inflation factor; *B*, unstandardized beta; SE, standard error.

Next, Model 3 included interaction terms between Event and all variables to identify whether the associations between predictors and finish time differed across Events. The inclusion of the interaction term with Event further significantly improved the model fit compared with Model 2 (Δ*R* ^2^ = 0.009, *F* of Δ*R* ^2^ = 5.2, *p* < 0.001, Table 2). As the primary objective of this model was to test interaction effects while controlling for all other variables, Type III ANOVA was performed to evaluate the unique contribution of each interaction term (Table S2). This analysis revealed significant main effects of PB, age, running distance, running frequency, and Event (running frequency: *p* = 0.005; the others: *p*s <0.001), whereas career, BMI, and longest run were not significant. Importantly, significant interaction effects were observed for the terms of PB × Event (*p* < 0.001, partial *η* ^2^ = 0.012), age × Event (*p* = 0.009, partial *η* ^2^ = 0.005), career × Event (*p* < 0.001, partial *η* ^2^ = 0.010), and BMI × Event (*p* < 0.001, partial *η* ^2^ = 0.011), whereas the terms of running distance × Event (*p* = 0.093), longest run × Event (*p* = 0.411), and running frequency × Event (*p* = 0.905) were not significant. These findings indicate that the relationships between either PB, age, career, or BMI and finish time differed depending on the level of Event, while the associations between either running distance, longest run, or running frequency and finish time were consistent across all Event groups.

### 3.4 Moderation effect of event

**FIGURE 2:**
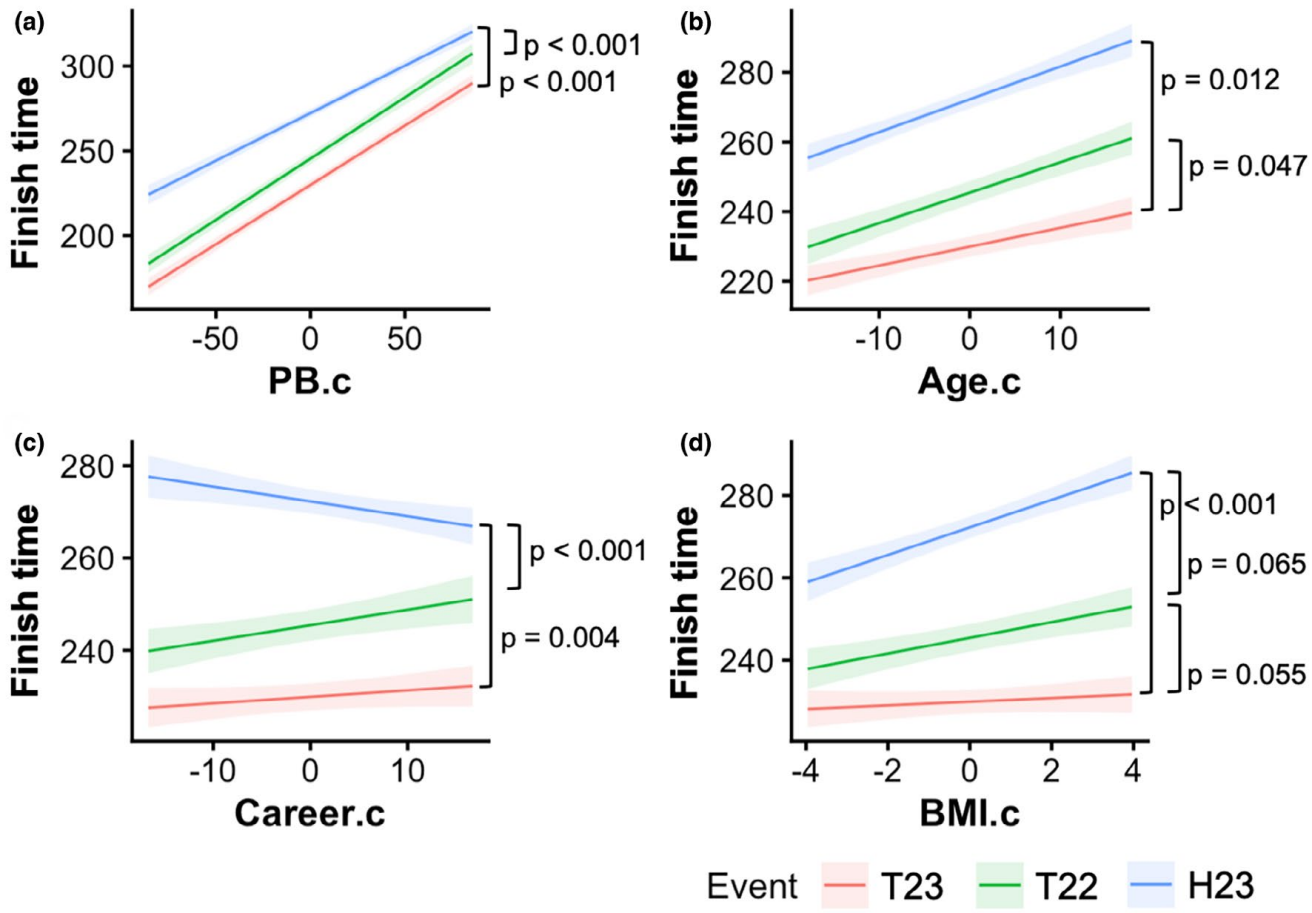
Significant interactions between variables and Event in predicting finish time. Interactions between Event and the following centered variables: (a) PB (PB.c); (b) Age (Age.c); (c) Career (Career.c); and (d) BMI (BMI.c). Regression lines were visualized across the range of each variable spanning ±2 SD from the mean. *p* values indicate pairwise comparison of slopes.

Subsequently, simple slope analyses were performed to further interpret the significant interaction effects identified in the Type III ANOVA by using estimated marginal trends (emtrends) from the emmeans package in R with Tukey adjustment for multiple comparisons. The results are illustrated in Figure 2 and summarized in Table S3. The emtrends indicated that PB was significantly associated with finish time across all event groups (T23: *B* = 0.70, SE = 0.03, *β* = 0.62; T22: *B* = 0.72, SE = 0.03, *β* = 0.64; H23: *B* = 0.56, SE = 0.03, *β* = 0.50, *ps* <0.001, Table S3A). Pairwise comparisons of slopes revealed that the slopes of PB were significantly steeper in T23 and T22 than in H23 (T23 vs. H23: Δ*B* = 0.14, SE = 0.04, Δ*β* = 0.12; T22 vs. H23: Δ*B* = 0.16, SE = 0.04, Δ*β* = 0.14, *ps* <0.001), while no significant difference was observed between T23 and T22 (*p* = 0.815) (Figure 2a and Table S3A). Age was also significantly associated with finish time across all event groups (T23: *B* = 0.54, SE = 0.10, *β* = 0.10; T22: *B* = 0.87, SE = 0.10, *β* = 0.16; H23: *B* = 0.94, SE = 0.10, *β* = 0.18, *ps* < 0.001, Table S3B). The slope in T22 and H23 was significantly more positive than that in T23 (T23 vs. T22: Δ*B* = −0.33, SE = 0.14, Δ*β* = −0.06, *p* = 0.047; T23 vs. H23: Δ*B* = −0.40, SE = 0.14, Δ*β* = −0.07, *p* = 0.012), while there was no significant difference between T22 and H23 (*p* = 0.883) (Figure 2b and Table S3B). The association between career and finish time was significant in T22 and H23 (T22: *B* = 0.34, SE = 0.11, *β* = 0.06, *p* = 0.003; H23: *β* = −0.32, SE = 0.10, *β* = −0.06, *p* = 0.002), but not in T23 (*p* = 0.156) (Table S3C). Pairwise comparisons of slopes confirmed that the slopes of career were significantly steeper in T23 and T22 than in H23 (T23 vs. H23: Δ*B* = 0.46, SE = 0.14, Δ*β* = 0.08, *p* = 0.004; T22 vs. H23: Δ*B* = 0.66, SE = 0.15, Δ*β* = 0.11, *p* < 0.001), while no significant difference was observed between T23 and T22 (*p* = 0.393) (Figure 2c and Table S3C). For BMI, simple slope analysis revealed a significant association with finish time in T22 and H23 (T22: *B* = 1.90, SE = 0.45, *β* = 0.08; H23: *B* = 3.34, SE = 0.46, *β* = 0.14, *ps* <0.001), whereas the association was not significant in T23 (*p* = 0.314) (Table S3D). Pairwise comparisons of slopes indicated a significantly steeper slope in H23 than in T23 (Δ*B* = −2.90, SE = 0.64, Δ*β* = −0.12, *p* < 0.001). Furthermore, while the comparisons between T23 and T22 (Δ*B* = −1.46, SE = 0.63, Δ*β* = −0.06, *p* = 0.055) and between T22 and H23 (Δ*B* = −1.44, SE = 0.64, Δ*β* = −0.06, *p* = 0.065) did not reach statistical significance, the pattern of results suggested a tendency for the slope to become progressively steeper across Event groups (Figure 2d and Table S3D). Overall, these results confirm that Event, which represents temperature differences, significantly moderates the associations between either PB, age, career, or BMI and finish time. The strength and significance of these associations varied depend on Event, as demonstrated by both simple slope analyses and pairwise comparisons of emtrends.

## 4 DISCUSSION

This study aimed to explore the key variables influencing the finish time of male runners in full‐marathons under hot environmental conditions. To this end, we examined variables potentially affecting finish time in marathon events held under different (low‐ to high‐) temperature conditions and identified the variables whose associations with finish time were moderated by the temperature differences between events. In this study, PB, age, career, BMI, running distance, longest run, and running frequency were collected via questionnaire as potential variables. The main findings were as follows: (1) Model 1, which excluded the Event variable, explained approximately 71% of the variance in finish time. In Model 2, which included the Event variable, the regression coefficients revealed a stepwise increase in the association between Event and finish time (T22 <T23 <H23). The inclusion of the Event variable significantly improved the model fit, increasing explained variance by 10.7%. (2) In Model 3, a significant moderating effect of Event was found in the relationship between finish time and either PB, age, career, or BMI, although the effect sizes were small. Specifically, for PB, slower PBs were associated with longer finish times across all Event groups, with a steeper slope observed in T23 and T22 compared with H23. For age, finish time became longer with increasing age across all Event groups, and this slope was steeper for T22 and H23 than for T23. For career, no significant association with finish time was observed in T23. In contrast, longer career was associated with longer finish time in T22 but with shorter finish time in H23. For BMI, higher BMI was associated with longer finish time in T22 and H23, and there was a tendency for the strength of this association to gradually increase from T23 (Event 1) to H23 (Event 3). (3) For the training variables, such as running distance, frequency, and longest run, no interactions with Event were observed. Running distance was not significantly associated with finish time in Model 1 but became significant after the inclusion of the Event variable in Model 2 and remained significant in Model 3. In contrast, the associations of longest run and running frequency with finish time observed in earlier models (Models 1 and 2) were attenuated after adjustment for Event and other predictors.

The present study treated the three marathon events as a categorical variable sorted in ascending order of temperature: Event 1 = T23 (mean Ta; 6.5°C; sWBGT: 11.2°C), Event 2 = T22 (mean Ta: 20.6°C; sWBGT; 22.2°C), Event 3 = H23 (mean Ta; 28.2°C; sWBGT: 31.5°C). The graded increase in finish time observed from Event 1 to 3 (Model 2 of Table 3) suggests that the effect of Event difference is not merely categorical but reflects a progressive negative impact on finish time along with rising temperature. The optimal temperature for full‐marathon performance has been generally reported to be approximately 10.0°C–12.5°C Ta (Ely, Cheuvront, & Montain, 2007; Martin & Buoncristiani, 1999; Maughan, 2010), or approximately 5.0°C–10.0°C WBGT (Ely, Cheuvront, Roberts, & Montain, 2007; Montain et al., 2007), across all runner types. However, slightly different ranges have been reported by some recent research. For example, the optimal Ta for elite and well‐trained athlete lies within a slightly higher range: 10°C–18.6°C (Mantzios et al., 2022; Scheer et al., 2021), while for runners with diverse running capacities, including recreational runners, the optimal Ta lies within a lower range (3.0°C–11.0°C Ta) (Arielle, 2025; El Helou et al., 2012). Importantly, marathon performance declines beyond the optimal ranges, leading to a gradual decrease in finish times or average race speeds. For the subjects of this study, Event 1 (T23) seems to have been held under environmental conditions close to optimum temperatures, while Events 2 (T22) and 3 (H23) were held under conditions in which the temperatures exceeded optimum levels, resulting in heat stress. According to the criteria of Mantzios et al. (2022), Events 1–3 could be classified as “Natural”, “Moderate heat”, and “Extreme heat” conditions, respectively (Mantzios et al., 2022). The marathon events examined in this study appear to be characterized by these conditions, and the following discussion is framed accordingly.

The addition of the Event variable significantly improved model fit, explaining an additional 10.7% of the variance in finish time (Table 2), and highlighting the importance of Event‐related temperature differences as a key factor influencing finish time. Furthermore, significant interactions were identified between Event and PB, age, career, or BMI (Figure 2 and Table S2), indicating that Event not only exerts a strong independent influence on finish time but also alters the pattern of associations between these predictors and finish time. Overall, these findings suggest that temperature difference functions as a structural determinant of finish time, both by directly affecting the magnitude of finish time and by modifying the strength of the associations between finish time and other variables. To date, numerous studies have identified factors associated with full‐marathon performance and attempted to construct prediction models for finish times using these factors (Alvero‐Cruz et al., 2020; Doherty et al., 2020; Keogh et al., 2019; Nikolaidis & Knechtle, 2024). The relationship between meteorological conditions, including Ta, and full‐marathon performance has also been energetically examined (Hodgson et al., 2022; Mantzios et al., 2022). However, to the best of our knowledge, no studies have systematically examined the combined effects of these predictive variables and Ta on predicting marathon performance. Our findings provide valuable data towards resolving this issue, and emphasize that Ta, and probably other environmental factors, should be carefully considered in the investigation of determinants of marathon performance.

Previous studies have reported that the thermal impact on marathon performance varies depending on the differences in running ability. The predominant view is that slower runners exhibit significantly reduced performance under higher heat conditions compared to faster elite runners, probably due to the longer duration of running, which exposes them to greater heat stress (Ely, Cheuvront, Roberts, & Montain, 2007; Montain et al., 2007; Vihma, 2010). By contrast, Gasparetto and Nesseler (2020) propose that faster elite runners perform high‐intensity exercise over shorter periods and consequently become more susceptible to the limiting effects of heat stress compared to slower runners, ultimately leading to a greater decline in performance (Gasparetto & Nesseler, 2020). The present study supports the latter view because the slope of the relationship between PB and finish time had no change in the moderate heat (T22) condition compared with the natural (T23) condition, but the slope became significantly flatter in the extreme heat (H23) condition (Figure 2a and Table S3A). This suggests that the relationship between running ability and marathon performance, such as a slower PB being associated with a longer finish time, is mitigated under extreme heat conditions. More simply, a faster runner might show a greater performance decline than a slower runner under such conditions.

For age, this study found that aging negatively relates to finish time regardless of temperature, and this tendency uniformly increases in environments exceeding the optimum temperature (Figure 2b and Table S3B). On the other hand, BMI is not associated with finish time at the optimal temperature; however, above the optimal temperature, BMI gains cause a gradual increase in the time, and this effect tends to become stronger with rising temperatures (Figure 2d and Table S3D). Previous studies have shown that marathon performance, evaluated based on finish time and race pace, peaks at around 35–39 years old, after which it declines with age even with vigorous training (Lehto, 2016; Lepers et al., 2021; Nikolaidis et al., 2019), and that the adverse effects of high temperatures (>15°C) on age‐related performance decline become more pronounced among 30–64 years old in male runners (Knechtle, Mcgrath, et al., 2021; Knechtle, Valero, et al., 2021). The older athlete's vulnerability to high temperature is widely accounted for by the age‐related declines in thermoregulatory capacity, especially in sweating and skin blood flow responses (Balmain et al., 2018; Kenney et al., 2021; Kenney & Munce, 2003). By contrast, it remains unclear how marathon performance varies with the combination of BMI and temperature. The finding that a steeper increase in finish time with increasing BMI and temperature (Figure 2d) might be helpful in partially explaining epidemiological data, in which higher BMI leads to higher risks of exertional heat stroke or illness (Gardner et al., 1996; Giersch et al., 2023). There is a supporting report indicating that individuals with higher BMIs experience greater cardiovascular strain during work in a hot and humid environment compared to those with lower BMIs (Dehghan et al., 2013). From the perspective of thermoregulation, our finding is consistent with previous investigations conferring a significant advantage to smaller subjects over larger subjects under heat‐stress conditions (Dennis & Noakes, 1999; Marino et al., 2000), while there are some inconsistent findings on the influence of body size in thermoregulatory responses (Foster et al., 2020). BMI is a simple and modifiable index derived from weight and height and may, therefore, represent a practical target for runners when developing training strategies for competitions where elevated temperatures are expected.

Interestingly, career had no relation to finish time under the natural temperature condition, whereas it was associated with finish time under the moderate and extreme heat conditions, under which the direction of association (positive or negative) differed depending on the condition (Figure 2c and Table S3C). Specifically, finish time became longer as career increased under the moderate heat condition (T22), whilst it became shorter as career increased under the extreme heat condition (H23). Years of running experience have been identified as a significant predictor of marathon performance (Bale et al., 1985; Keogh et al., 2020), likely reflecting accumulated physiological adaptations and improved racing strategies developed through long‐term training. Our findings suggest that longer running experience may be a key factor in achieving shorter finish times at summer races where the Ta are extremely high.

In a recent systematic review, a negative relationship has been found between training status and marathon performance, whereby increases in each training parameter led to shorter marathon finish times (Doherty et al., 2020). The present study also found a similar trend in running distance and frequency, with no significant interaction between the training variables and temperature (Table 2 and Table S2). These results indicate that the strategy of increasing training volume and frequency is effective for enhancing marathon performance, and this benefit is conferred independent of the temperature. However, in view of the prior study on heat acclimation, it cannot be assumed that training behavior and temperature‐dependent changes in marathon performance are entirely unrelated. For example, prior aerobic training in hot environments has been shown to induce heat acclimation, thereby enhancing endurance performance under similar conditions, primarily through increases in plasma volume, and improvements in perspiration function and cardiovascular stability (James et al., 2017; Mikkelsen et al., 2019; Périard et al., 2024).

All participants volunteered to take part in the study, resulting in differences in participant characteristics across the marathon races, except for age. Broadly speaking, compared to participants in T22 and T23, those in H23 tended to have longer PB, higher BMI, longer career, shorter running distance, and lower running frequency (Table 1). As the questionnaire‐based measurements were obtained through self‐report, the possibility that discrepancies between actual and self‐reported values affected the present findings cannot be ruled out. Indeed, previous studies have reported that recreational runners and endurance athletes tend to slightly underreport body mass and overreport body height, resulting in an underestimation of BMI (Knechtle et al., 2012; Nikolaidis & Knechtle, 2020). However, these discrepancies are relatively small and smaller than the self‐report bias reported in the general population (Nikolaidis & Knechtle, 2020). Therefore, although caution is required when interpreting the self‐reported data, the data obtained in this study may have a certain degree of reliability. Moreover, because this study examined the impact of rising temperatures using two marathon events held in different seasons and at different venues, the possibility that course differences influenced the results cannot be ruled out.

The present study examined the effects of temperature on the relationships between each variable and finish time across three categorical conditions: “Natural”, “Moderate heat”, and “Extreme heat”. However, the participants were predominantly Japanese male recreational runners, and the effects of heat were estimated mainly from a single warm‐weather marathon event (H23) conducted at one location and time. Thus, the findings were derived from a specific runner population and a relatively narrow range of environmental conditions, which may limit their generalizability to other marathon events and populations, including female runners and elite athletes. Future research should examine these relationships in more diverse runner populations and across a broader range of WBGT values, including genuinely hot and cool conditions.

## 5 CONCLUSION

The present study found that, among the variables which can be accessed by a questionnaire survey, running ability (best marathon time), age, years of running experience, and BMI were associated with full‐marathon finish time and were also susceptible to the influence of temperature differences, especially of temperature gains. Specifically speaking, it was observed that longer finish times due to hot weather tended to be more pronounced in runners with higher running ability, older age, less running experience, and higher BMI. In contrast, training status, such as weekly running distance and frequency, was related to the finish time, yet unaffected by the change of temperature. Taken together, the findings of this study indicate that age, an irreversible factor, and running ability, running experience, and BMI, which are modifiable factors, could be major targets for strategies to combat hot weather during the marathon race. These findings are likely to prove valuable for runners and coaches as they draw up full‐marathon training and conditioning plans in the future, when rising temperatures resulting from global warming are expected.

## AUTHOR CONTRIBUTIONS

**Koshiro Inoue:** Conceptualization; data curation; formal analysis; funding acquisition; investigation; methodology; validation; visualization. **Takemune Fukuie:** Investigation. **Akihiko Yamaguchi:** Project administration; supervision. **Yoshiharu Nabekura:** Investigation; project administration; resources. **Toru Ishihara:** Investigation; methodology.

## FUNDING INFORMATION

This work was supported in part by the Japan Society for the Promotion of Science Grants‐in‐Aid for Scientific Research C (23K10637) (to K.I.).

## CONFLICT OF INTEREST STATEMENT

No conflicts of interest, financial or otherwise, are declared by the authors.

## ETHICS STATEMENT

This study was conducted in accordance with the Declaration of Helsinki and approved by the Institutional Ethics Committee of Health Sciences University of Hokkaido (approved no. 22R191189, 23R212216).

## Supporting information


**Table S1:** Univariate associations between variables and finish time.

**Table S2:** Type III ANOVA results for Model 3.

**Table S3:** Simple slope analyses and pairwise comparisons of slopes across events.

## Data Availability

The data generated and analyzed during this study are available from the corresponding author on reasonable request.
