# Supplementary material for: Temperature‐driven modulation of factors influencing full‐Marathon time in male recreational runners
Source: Physiol Rep. 2026 Sep 8;14(17):e71096. doi: 10.14814/phy2.71096 (PMC13554990; doi:10.14814/phy2.71096)
Supplement: Supplementary file 1 [file PHY2-14-e71096-s001.docx]

**Supplementary Table 2 Type III ANOVA results for Model 3**

| Variable | df | F | *p*-value | Partial *η²* |
| --- | --- | --- | --- | --- |
| Intercept | 1 | 2020.53 | < 0.001 | 0.514 |
| PB | 1 | 777.06 | < 0.001 | 0.289 |
| Age | 1 | 31.03 | < 0.001 | 0.016 |
| Career | 1 | 2.02 | 0.156 | 0.001 |
| BMI | 1 | 1.02 | 0.314 | 0.001 |
| Running distance | 1 | 14.95 | < 0.001 | 0.008 |
| Longest run | 1 | 0.76 | 0.383 | 0.000 |
| Running frequency | 3 | 4.31 | 0.005 | 0.007 |
| Event | 2 | 17.11 | < 0.001 | 0.018 |
| PB × Event | 2 | 11.23 | < 0.001 | 0.012 |
| Age × Event | 2 | 4.72 | 0.009 | 0.005 |
| Career × Event | 2 | 10.11 | < 0.001 | 0.010 |
| BMI × Event | 2 | 10.32 | < 0.001 | 0.011 |
| Running distance × Event | 2 | 2.37 | 0.093 | 0.002 |
| Longest run × Event | 2 | 0.89 | 0.411 | 0.001 |
| Running frequency × Event | 6 | 0.36 | 0.905 | 0.001 |
