# Supplementary material for: Temperature‐driven modulation of factors influencing full‐Marathon time in male recreational runners
Source: Physiol Rep. 2026 Sep 8;14(17):e71096. doi: 10.14814/phy2.71096 (PMC13554990; doi:10.14814/phy2.71096)
Supplement: Supplementary file 2 [file PHY2-14-e71096-s002.docx]

**Supplementary Table 1 Univariate associations between variables and finish time**

| Variable | β | 95% CI | *p*-value |
| --- | --- | --- | --- |
| PB | 0.82 | 0.79, 0.84 | < 0.001 |
| Age | 0.24 | 0.19, 0.28 | < 0.001 |
| Career | 0.06 | 0.02, 0.11 | 0.006 |
| BMI | 0.42 | 0.38, 0.46 | < 0.001 |
| Running distance | -0.55 | -0.59, -0.51 | < 0.001 |
| Longest run | -0.30 | -0.34, -0.26 | < 0.001 |
| Running frequency |  |  |  |
| Group 1: < 1 | Reference |  |  |
| Group 2: 1-2 | -0.49 | -0.74, -0.25 | < 0.001 |
| Group 3: 3-4 | -1.17 | -1.41, -0.93 | < 0.001 |
| Group 4: ≧ 5 | -1.75 | -2.00, -1.51 | < 0.001 |
| Event |  |  |  |
| 1: T23 | Reference |  |  |
| 2: T22 | 0.22 | 0.13, 0.31 | < 0.001 |
| 3: H23 | 1.19 | 1.09, 1.29 | < 0.001 |

Regression coefficients (standardized β) are shown for each variable. PB: personal best; Career: years of running experience; BMI: body mass index.
