# Supplementary material for: Temperature‐driven modulation of factors influencing full‐Marathon time in male recreational runners
Source: Physiol Rep. 2026 Sep 8;14(17):e71096. doi: 10.14814/phy2.71096 (PMC13554990; doi:10.14814/phy2.71096)
Supplement: Supplementary file 3 [file PHY2-14-e71096-s003.docx]

**Supplementary Table 3 Simple slope analyses and pairwise comparisons of slopes across events**

| Simple slope analysis | | | | | |  | Pairwise comparison | | | | | |
| --- | --- | --- | --- | --- | --- | --- | --- | --- | --- | --- | --- | --- |
| Event | B | SE | β | t | *p*-value |  | Pair | ΔB | SE | Δβ | t | *p*-value |
| A. PB |  |  |  |  |  |  |  |  |  |  |  |  |
| 1: T23 | 0.70 | 0.03 | 0.62 | 27.88 | < 0.001 |  | 1 vs 2 | -0.02 | 0.04 | -0.02 | -0.61 | 0.815 |
| 2: T22 | 0.72 | 0.03 | 0.64 | 26.60 | < 0.001 |  | 1 vs 3 | 0.14 | 0.04 | 0.12 | 3.86 | < 0.001 |
| 3: H23 | 0.56 | 0.03 | 0.50 | 21.47 | < 0.001 |  | 2 vs 3 | 0.16 | 0.04 | 0.14 | 4.31 | < 0.001 |
| B. Age |  |  |  |  |  |  |  |  |  |  |  |  |
| 1: T23 | 0.54 | 0.10 | 0.10 | 5.57 | < 0.001 |  | 1 vs 2 | -0.33 | 0.14 | -0.06 | -2.37 | 0.047 |
| 2: T22 | 0.87 | 0.10 | 0.16 | 8.65 | < 0.001 |  | 1 vs 3 | -0.40 | 0.14 | -0.07 | -2.85 | 0.012 |
| 3: H23 | 0.94 | 0.10 | 0.18 | 9.26 | < 0.001 |  | 2 vs 3 | -0.07 | 0.14 | -0.01 | -0.48 | 0.883 |
| C. Career |  |  |  |  |  |  |  |  |  |  |  |  |
| 1: T23 | 0.14 | 0.10 | 0.02 | 1.42 | 0.156 |  | 1 vs 2 | -0.20 | 0.15 | -0.03 | -1.31 | 0.393 |
| 2: T22 | 0.34 | 0.11 | 0.06 | 3.00 | 0.003 |  | 1 vs 3 | 0.46 | 0.14 | 0.08 | 3.23 | 0.004 |
| 3: H23 | -0.32 | 0.10 | -0.06 | -3.11 | 0.002 |  | 2 vs 3 | 0.66 | 0.15 | 0.11 | 4.31 | < 0.001 |
| D. BMI |  |  |  |  |  |  |  |  |  |  |  |  |
| 1: T23 | 0.45 | 0.44 | 0.02 | 1.01 | 0.314 |  | 1 vs 2 | -1.46 | 0.63 | -0.06 | -2.31 | 0.055 |
| 2: T22 | 1.90 | 0.45 | 0.08 | 4.22 | < 0.001 |  | 1 vs 3 | -2.90 | 0.64 | -0.12 | -4.54 | < 0.001 |
| 3: H23 | 3.34 | 0.46 | 0.14 | 7.25 | < 0.001 |  | 2 vs 3 | -1.44 | 0.64 | -0.06 | -2.24 | 0.065 |

Simple slope analyses of variables showing significant interactions with Event when predicting finish time and pairwise comparisons of slopes between Events.
